# Do the benefits continue? Long term impacts of the Anatomy Education Research Institute (AERI) 2017

**DOI:** 10.1186/s12909-022-03883-w

**Published:** 2022-11-24

**Authors:** Polly R. Husmann, James J. Brokaw, Valerie Dean O’Loughlin

**Affiliations:** 1 Medical Sciences Program, Indiana University School of Medicine, 2631 E Discovery Parkway, Bloomington, IN 47408 USA; 2 Department of Anatomy, Cell Biology & Physiology, Indiana University School of Medicine, Indianapolis, IN USA

**Keywords:** Education research, Faculty development, Community of practice, SOTL, Scholarship of teaching and learning, Medical education

## Abstract

**Background:**

The Anatomy Education Research Institute (AERI) was held in Bloomington, Indiana in July of 2017. Previous research has shown that AERI was successful in meeting Kirkpatrick’s first two levels of evaluation via positive initial reactions and learning gains identified at the end of AERI. This manuscript demonstrates continued success in Kirkpatrick levels two and three via six-month and thirty-month follow-up surveys and nine-month follow-up focus groups and interviews.

**Methods:**

Quantitative analyses were completed using Microsoft Excel (2019) and SPSS version 26 while qualitative analyses were completed for both survey responses and focus groups/interviews using thematic analyses.

**Results:**

Results demonstrate that the learning gains seen immediately post-AERI 2017 were sustained for all participants (accepted applicants and invited speakers). Qualitative results continued to demonstrate positive reactions to AERI 2017. Both quantitative and qualitative results demonstrated that the main obstacle to educational research for most participants is time, while collaboration, IRB, institutional roadblocks, and devaluing of educational research were also identified as obstacles.

**Conclusions:**

The research presented here indicates positive outcomes to Kirkpatrick Levels 1, 2, & 3 of evaluation following AERI 2017. However, substantial obstacles still exist for researchers in medical education. The need for a sustained community of practice for educational researchers was suggested as a potential buffer against these obstacles and multiple options for providing that community are discussed.

**Supplementary Information:**

The online version contains supplementary material available at 10.1186/s12909-022-03883-w.

## Background

The Anatomy Education Research Institute (AERI) was held in 2017 at the campus of Indiana University, Bloomington with the express purpose of pairing individuals experienced in anatomy education research with novices who could bring new backgrounds and ideas to the field. To that end, the institute was funded by an Innovations grant from the American Association of Anatomists (now the American Association for Anatomy – both referred to as AAA) and included five days of plenaries, workshops, and small group mentoring. The organizers have used Kirkpatrick’s Four Levels of Evaluation Model [1, 2] to evaluate the impact of this institute. These levels include 1) Reaction (i.e., participants’ thoughts or reactions to the institute), 2) Learning (evidence of increasing participant knowledge from the institute), 3) Behavior (changes to participants’ behavior following the institute), and 4) Results (ultimate outcomes associated with participants’ increased knowledge and changed behavior following the institute). Our previous publications have focused on the first level of evaluation (reaction) during AERI 2017 [3] and the second level of evaluation (learning) immediately following AERI 2017 [4]. This report continues that evaluation by examining the maintenance of those attitudes and learning gains (Kirkpatrick levels 1 & 2) and assessing whether participants’ behavior was impacted by this experience (Kirkpatrick level 3).

### Previous research on the Anatomy Education Research Institute (AERI) 2017

O’Loughlin et al. [3] described the development of AERI 2017, including the justification for the set-up, the daily schedule, and the development of the instruments that would be used to assess the institute’s effectiveness. The article summarized the background literature that supported the development of an institute that focused on more than just improving teaching skills [5–8] and discussed the other opportunities for this type of professional development currently available through various schools or organizations (e.g., references [9–12]). That work then went on to explain participants’ initial reactions to various parts of the institute such as length, scheduling, organization, etc. Finally, O’Loughlin et al. [3] described the active Twitter participation of invited speakers, accepted applicants, and AERI non-participants during the institute and the potential future options for sustaining AERI.

Husmann et al. [4] expanded on this research to discuss the learning gains (Kirkpatrick level 2) that resulted immediately following AERI 2017 as seen on the pre-AERI and post-AERI surveys. Increased knowledge of anatomy education-related resources and topics was seen for both invited speakers and accepted applicants, further supporting Steinert et al.’s conclusions that mentors can also benefit from mentoring others [8, 13, 14]. Qualitative analyses also indicated the need for educational researchers to have a community of practice to support their work as well as resources, such as time, funding, and the respect and buy-in of their colleagues and administrations. Only time would tell how these ideas would continue to evolve when the participants returned to their home institutions.

Building on these studies, the present manuscript will focus on the longer-term effects from AERI 2017. In particular, this work will address the following research questions:

- How do the learning gains seen immediately following AERI compare to surveys completed six months later?
- How much progress have participants (accepted applicants and invited speakers) made on the three goals that they set during AERI 2017 after six months and after thirty months?
  o Corollary: What other teaching and/or educational research activities have participants completed since attending AERI 2017?
- How do participants’ (accepted applicants and invited speakers) perceptions of AERI change six to nine months and thirty months after they return to their home institutions?
- What obstacles have participants encountered since attending AERI and what types of support might be beneficial in overcoming these obstacles?

## Methods

Previous research has demonstrated the need for multiple data sources, including both quantitative and qualitative methods, to be utilized in assessing outcomes associated with faculty development initiatives [7, 14]. As such, the AERI 2017 organizers developed and administered multiple survey instruments as well as focus groups and individual interviews. This manuscript will focus on the six-month and thirty-month follow-up surveys and the nine-month follow-up focus groups and interviews with comparisons to the surveys that were administered at the beginning of the institute and at the end of the institute (these surveys and results have been previously described in O’Loughlin et al. [3] and Husmann et al. [4]). All AERI surveys and interviews were completed in accordance with Indiana University Institutional Review Board (IRB) protocol #1704969308.

### Six-month follow-up survey

The six-month follow-up survey (like the pre-AERI and post-AERI surveys) was modeled from previous surveys that had been utilized by the American Physiological Society’s Institute for Teaching and Learning (APS-ITL), which was first held in 2014 and offered every other year thereafter. All surveys were linked using a randomly assigned three-digit number that was printed on the participants’ (invited speakers and accepted applicants) name tags. For the six-month survey, this three-digit number was then linked to their e-mail address so that the number would auto-populate when the participant logged into the survey. The survey consisted of seven blocks. The first block included open-ended questions asking participants to list up to ten words that they associate with educational research, up to three obstacles that they have encountered to education research, other ideas for additional topics that should be discussed at AERI, etc. Block two then asked participants about their knowledge of books and journals related to the field of anatomy education on a six-point Likert scale. Block three also used a six-point Likert scale to ask participants about the educational scholarship activities that they had taken part in since AERI 2017. Blocks 4–6 then asked participants about their progress on the goals that they had created during AERI 2017. This was done by pre-populating the goals that they had input to the post-AERI survey into each block and then asking the participant if they had started the goal (or why not), if they had completed the goal (or why not), and if they still planned to complete the goal (and if so, when). Finally, the last block of the survey asked for any final comments on AERI 2017 and if the participant would be willing to complete a follow-up interview.

The six-month follow up survey was administered using Qualtrics (Qualtrics, Provo, UT). E-mails inviting all participants to complete the survey were sent out through the Qualtrics program, followed by four reminder e-mails to any unfinished respondents over the course of two and a half weeks. For further information on the development of this survey and its predecessors, please see O’Loughlin et al. [3] and Husmann et al. [4].

### Focus groups and interviews

All AERI participants (i.e., speakers and accepted applicants) were invited (via email) to participate in a focus group at the 2018 Experimental Biology annual meeting, which was approximately nine months after the completion of AERI 2017. Seven speakers and nine accepted applicants attended the focus group at the Experimental Biology meeting. Two AERI attendees (one speaker and one accepted applicant) were not able to attend the focus group but were willing to be interviewed. These individuals were interviewed separately by one of the authors (V.D.O.) and each of the interviews was audio recorded. The same questions asked of the focus groups also were asked of the interviewees.

The focus group was split into two groups – one for invited speakers and one for accepted applicants. The group for invited speakers was led by V.D.O. while the group for accepted applicants was co-led by J.J.B. and P.R.H. Both groups used a semi-structured format with a set list of questions that were developed by all three authors in advance based on results from the previous surveys. For this manuscript, we will focus on the following two questions, as they provided the richest data:

1. What have you found beneficial from participating in AERI 2017?
2. What are your obstacles to performing educational research – are they different from what you mentioned on the 6^th^ month survey? Is there anything that AAA or AERI could do to help with those obstacles?

We also briefly discuss the question “What are some things we could do to improve future versions of AERI?” for the purposes of quality improvement. Additional questions were then asked based on topics and ideas presented by the participants. Both focus groups were audio recorded from two different locations in the group to ensure all voices were recorded clearly.

All audio recordings were transcribed by J.J.B. In the transcriptions, individuals were identified in the transcript only as “AERI co-organizer” or “interviewee.” A different individual (V.D.O) was responsible for analyzing the transcriptions, using a qualitative thematic approach (described below).

### Thirty-month follow-up survey

The thirty-month follow-up survey was again modeled from previous surveys that had been used in connection with AERI 2017. Surveys were still linked using their randomly assigned three-digit number, which was again set to auto-populate from the participant’s e-mail address. The survey consisted of four blocks. The first block included open-ended questions asking participants what they now felt had been the most helpful or useful part of their AERI experience and asking what obstacles they have encountered to education research since participating in AERI 2017. Block two then asked participants about the teaching and educational research activities in which they had participated since AERI 2017. Block three focused on which of the three goals set at AERI they had started, which they had completed, and what were the obstacles or outcomes of those projects. Specifically, the participants were again asked if they had started the goal (or why not), if they had completed the goal (or why not), and if they still planned to complete the goal (and if so, when). Finally, the last block of the survey asked participants to upload a current copy of their curriculum vitae and asked for any final comments on AERI 2017.

The thirty-month follow-up survey was again administered using Qualtrics (Qualtrics, Provo, UT). E-mails inviting all participants to complete the survey were sent out through the Qualtrics program on February 18, 2020, followed by reminder e-mails to any unfinished respondents over the course of three weeks with the final reminder (and final responses received) on March 10, 2020.

### Quantitative analysis

All quantitative analysis was completed using SPSS version 26 (IBM Corp, Armonk, NY). Descriptive statistics are reported, however inferential statistics were not utilized due to the limited sample. Figures were created using Microsoft Excel (2019).

### Qualitative analysis

Before discussing the qualitative data, it is important for us (the authors) to present our reflexivity statement, whereby we acknowledge how our roles in AERI, our beliefs about the value of education research, and our life experiences may impact our evaluation of this data. As the co-organizers of AERI, the authors strongly believe in the value of anatomy education research and how well-developed projects can positively influence the field. We have trained anatomy graduate students to become education researchers, so we recognize that this experience may influence how we evaluate the training of participants at AERI. We were further along an 'education research’' path than the AERI participants, but we recognize that we as well as the participants are traversing along a similar path. We believe our experiences and prior life situations allowed us insight when digging into this data, yet at the same time, we tried to be mindful of the fact that, as the AERI co-organizers, we had a vested interest in seeing long-term positive effects from the Institute. The qualitative analysis of the open-ended survey responses was completed by a single author (P.R.H.). This coding was completed using an inductive thematic model [15]. Answers to each question were read multiple times to establish familiarity with the content. Initial coding was then completed for each question and codes that became unwieldy were divided into subcodes. Overarching codes were also condensed into larger themes for interpretation. Codes were also counted for comparisons between invited speakers and accepted applicants and for comparisons with the data from pre-AERI and/or post-AERI surveys [4]. Some responses were dual-coded if multiple ideas applied. As such, results will occasionally show more codes than the listed number of responses. It should be noted that some qualitative researchers recommend that codes not be counted, as this is a misguided attempt to quantify the qualitative data, rather than focus on the richness of the data. However, as some of the survey questions asked individuals to list a specific number (e.g., three obstacles in pursuing their research, ten words they thought of to describe education research), quantification of said obstacles and terms is acceptable as corroborative counting to support the triangulation of qualitative and quantitative analyses [16].

The qualitative analysis of the focus groups and interview transcripts were completed solely by another author (V.D.O.). This task was intentionally assigned to a different author to remove the potential for personal bias (that may have resulted from coding the surveys) when determining codes for this new data set (focus groups and interviews). As with the survey responses, an inductive thematic approach was used in evaluating the transcripts, with some minor differences. First, the interview transcripts were matched with their respective AERI group – the single AERI speaker interview transcript was reviewed alongside the speaker focus group, while the single accepted applicant interview was reviewed alongside the accepted applicant focus group transcript. All transcripts were read in their entirety to establish the dynamic and flow of the focus groups and the interview sessions. Responses to each question were read multiple times, to determine the initial codes for each group. Codes were edited and refined through each reading, and themes were developed for codes that were aligned (or opposed) in some fashion. Codes also were compared between AERI speaker and participant groups, to determine if common codes or themes were present. The authors did not tabulate the frequency of the codes, as some participants were more vocal than others, and a code count would skew or inflate individual opinions. In this case, as previous qualitative researchers have mentioned, a quantification of codes would not be appropriate [15].

## Results

### Survey

**Fig. 1:**
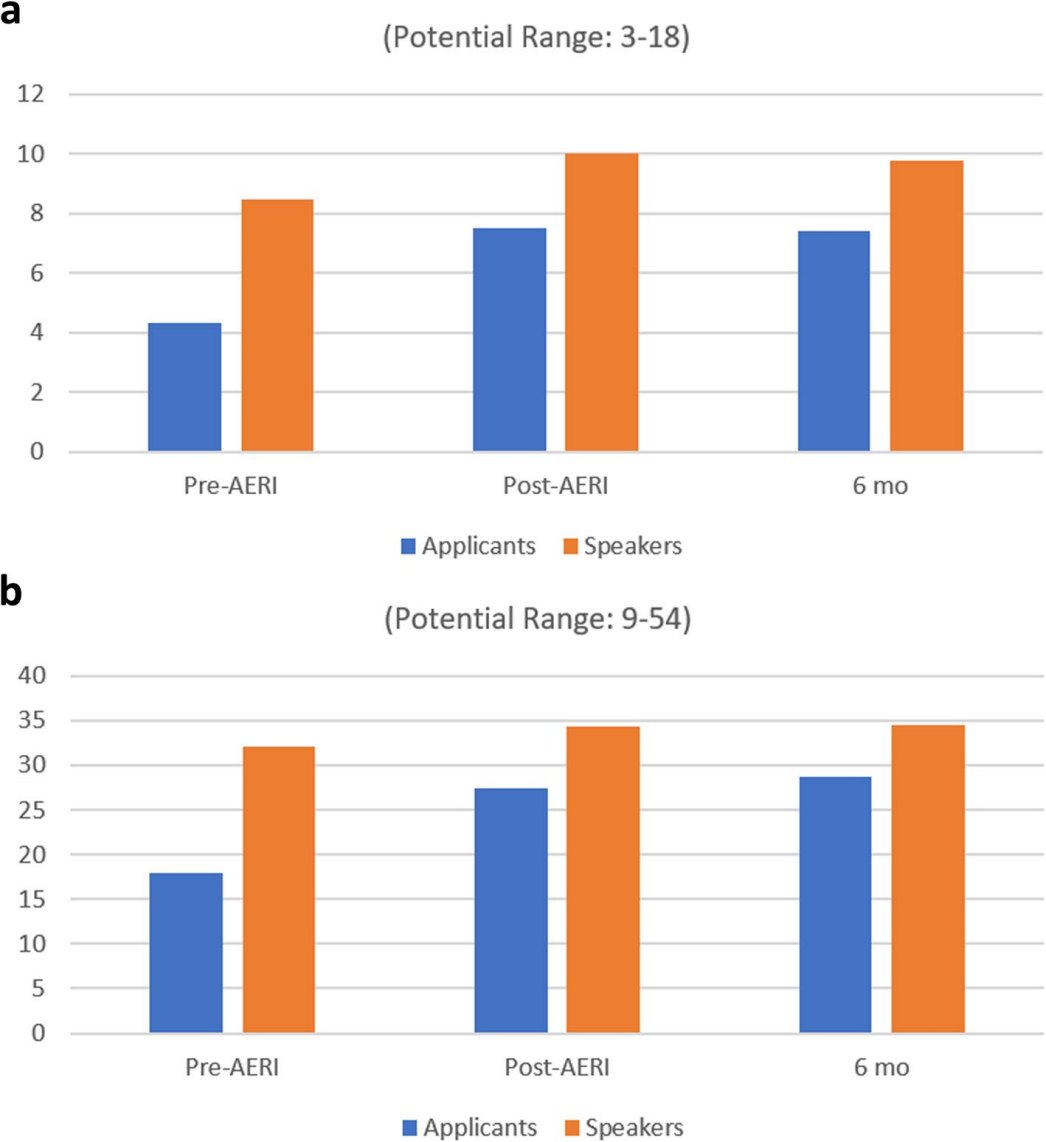
Knowledge gains associated with the Anatomy Education Research Institute (AERI) 2017: a) texts, b) journals

Twenty-four AERI 2017 participants (38.7%) completed the six-month follow-up survey; fifteen who were accepted applicants and nine who were invited speakers. Data on knowledge gains following AERI 2017 demonstrated maintenance and even some continued increase following the institute. Knowledge of texts related to anatomy education research demonstrated that the gains seen previously on the post-AERI surveys [4] were maintained for at least six months for both applicants and speakers (Fig. 1a). Data on knowledge of journals related to anatomy education research were likewise sustained following AERI 2017 (Fig. 1b).

**Fig. 2:**
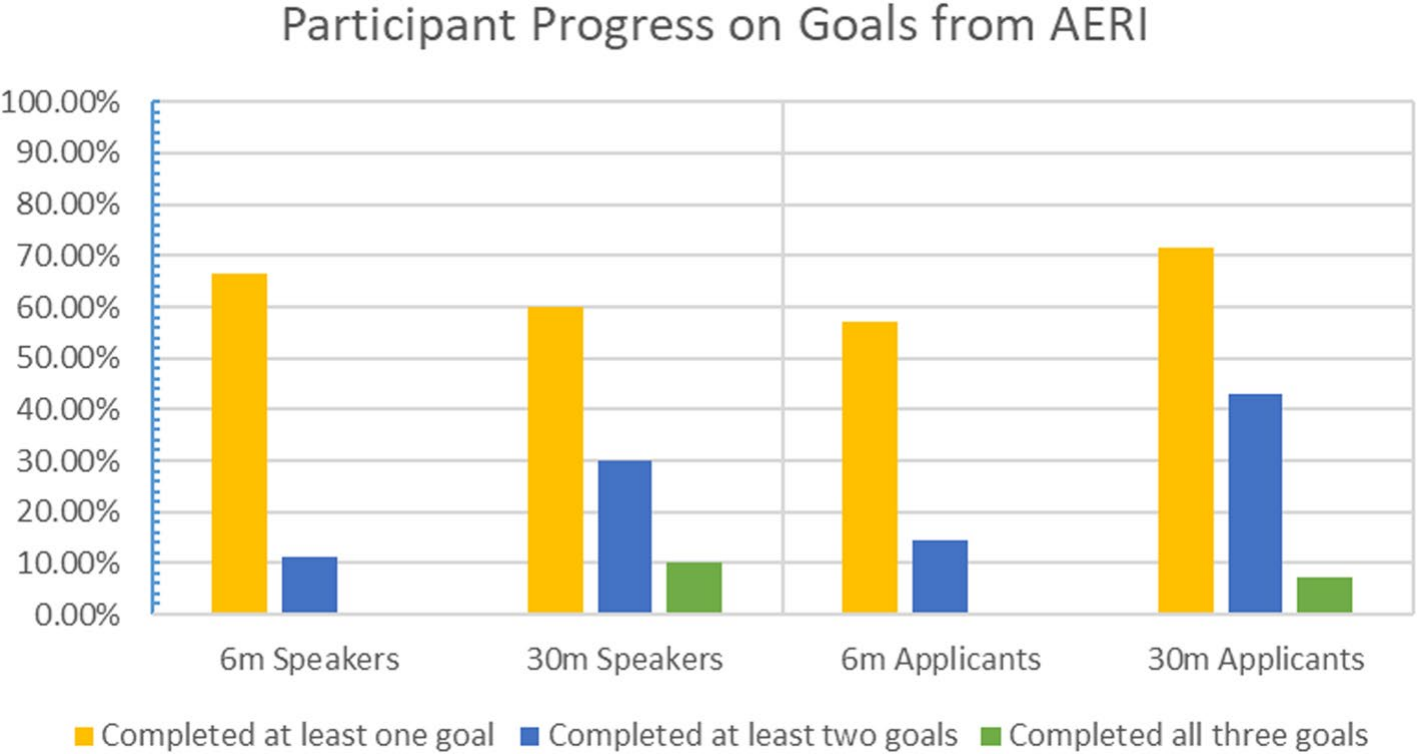
Participant Progress on Goals Established at the Anatomy Education Research Institute (AERI) 2017

When participant follow-up on goals from AERI 2017 were evaluated (Fig. 2), it was found that 91.3% of participants started work on their first goal within six months. In addition, 78.3% of respondents had started their second goal and 70% had even started work on their third goal. When analyzing goal completion, 28.6% stated that they had completed their first goal with the same proportion also having completed their second goal. For their third goal, 25% of respondents reported completion. For the goals that were not either started or completed, participants still planned to complete 80% of these goals with common reasons for lack of completion including teaching loads, changing roles, or lack of time up to that point.

The open-ended survey questions also yielded a wide variety of data. The obstacles that our participants have run into are shown in Table 1 with comparisons to the previous pre-AERI and immediately post-AERI survey data. The obstacles to education research that were most cited by both applicants and speakers were *time* and the *need for collaboration*. These were expressed even more commonly in the six-month follow-up survey than had been seen on either the pre-AERI or post-AERI surveys. Additional codes that saw increased usage since the post-AERI survey include *IRB approval* and *deciding among multiple projects*. On a positive note, all codes indicating concerns about educational research (e.g., *controls*, *quality*) continued to decline in prevalence.

**Table 1:** Participant perceptions of obstacles to educational research

Themes	Codes^b^	Accepted Applicants			Invited Speakers			Total (all AERI participants)			Change in frequency of codes between surveys
		Number of coded responses on Pre-AERI survey (*n* = 91)	Number of coded responses on Post-AERI survey (*n* = 84)	Number of coded responses on 6 month survey (*n* = 30)	Number of coded responses on Pre-AERI survey (*n* = 50)	Number of coded responses on Post-AERI survey (*n* = 53)	Number of coded responses on 6 month survey (*n* = 18)	Number of coded responses on Pre-AERI survey (*n* = 141)	Number of coded responses on Post-AERI survey (*n* = 144)^a^	Number of coded responses on 6 month survey (*n* = 48)	
Community needs	Time	8 (8.8%)	20 (23.8%)	14 (46.7%)	6 (12.0%)	11 (20.7%)	7 (38.9%)	14 (9.9%)	31 (21.5%)	21 (43.8%)	Increase
	Money	13 (14.3)	9 (10.7)	2 (6.7)	9 (18.0)	10 (18.9)	2 (11.1)	22 (15.6)	20^a^ (13.9)	4 (8.3)	Decrease
	Respect/ support	18 (19.8)	22 (26.2)	6 (20)	12 (24.0)	14 (26.4)	1 (5.6)	30 (21.3)	38^a^ (26.4)	7 (14.6)	Increase, decrease
	Collaboration	4 (4.4)	6 (7.1)	4 (13.3)	3 (6.0)	4 (7.5)	3 (16.7)	7 (5.0)	10 (6.9)	14 (29.2)	Increase
	IRB	0	0	1 (3.3)	0	0	1 (5.6)	0	0	2 (4.2)	Increase
Concerns about educational research	Background Knowledge	22 (24.2)	16 (19.0)	1 (3.3)	3 (6.0)	6 (11.3)	1 (5.6)	25 (17.7)	24^a^ (16.7)	2 (4.2)	Decrease
	Controls	15 (16.5)	8 (9.5)	0	11 (22.0)	4 (7.5)	0	26 (18.4)	15^a^ (10.4)	0	Decrease
	Quality	3 (3.3)	1 (1.2)	0	7 (14.0)	2 (3.8)	0	10 (7.1)	3 (2.1)	0	Decrease
	Participation	9 (9.9)	5 (5.9)	1 (3.3)	2 (4.0)	4 (7.5)	0	11 (7.8)	9 (6.2)	1 (2.1)	Decrease
Options	Deciding among multiple project	0	0	1 (3.3)	0	0	3 (16.7)	0	0	4 (8.3)	Increase

^a^Some individuals entered an incorrect ID number so their applicant/speaker status is unknown

^b^The pre-conference survey received 154 obstacle responses, of which 141 (91.5%) were coded with at least one code. The post-conference survey received 149 obstacle responses, of which 144 (96.6%) were coded with at least one code. Both pre-conference and post-conference survey data from Husmann et al. [4]

When considering the ten words that participants associated with education research, the total results along with their comparisons from the pre-AERI and post-AERI surveys may be seen in Table 2. Particular trends of note include a slight decrease in use of the code for *difficulty* and a slight increase in words associated with *non-rigorous perceptions* of educational research. However, both codes for *important* and *innovative* continued to increase even since the post-AERI survey. Finally, the ideas of *community* decreased substantially since the post-AERI survey while the codes for *lack of respect* and *funding* increased.

**Table 2:**
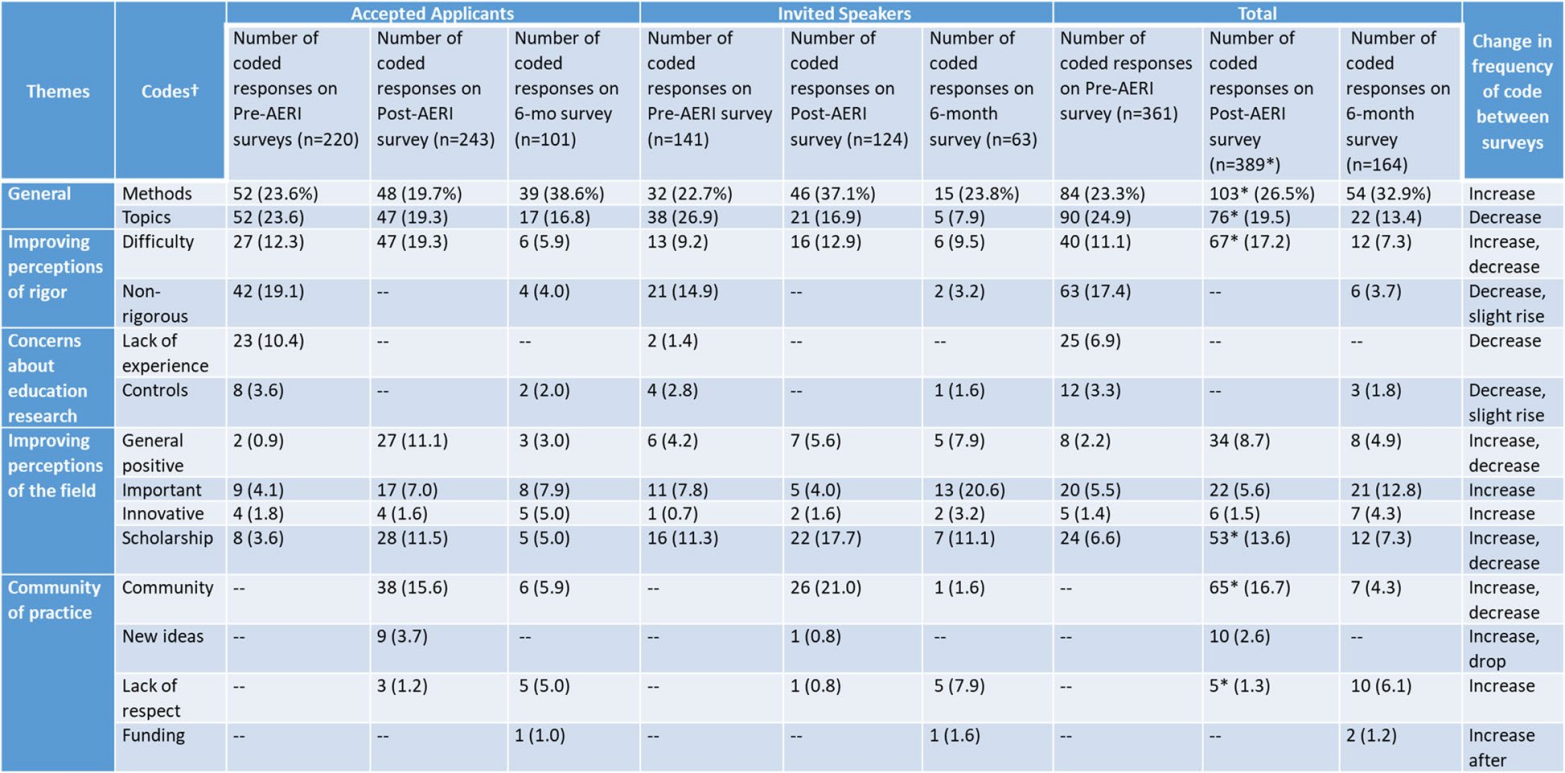
Thematic analysis of the ten words that participants associated with education research on the pre-AERI, post-AERI, and six-month follow-up surveys

### Focus groups and interviews

As previously mentioned, the focus group and interview data included 10 accepted applicants and 8 invited speakers. Results are listed below.

#### Perceived benefits of the Anatomy Educational Research Institute (AERI)

Table 3 lists the themes and codes that emerged from responses to question 1: “What have you found beneficial from participating in AERI?” Codes and themes were compared and contrasted between the accepted applicant group (A) and the invited speaker/mentor group (S) and are discussed in detail in the following paragraphs.

**Table 3:** Thematic Analysis Codebook for “What did you find most beneficial about AERI?

Accepted Applicants (A)		Speakers (S)	
**Theme**	**Subtheme**	**Theme**	**Subtheme**
Community		Community	
Collaboration	With other mentees With mentors With other twitter followers	Collaboration	With mentees With other mentors Multi-institutional collaborations
AERI’s organization: it helped them to:	Focus Goal-set Organize their workflow for success	AERI’s organization: it helped them to:	Focus Goal-set Organize their workflow for success
Motivational/inspiring: it encouraged them to:	Expand their knowledge base Complete their projects Present and publish their educational research	Motivational/inspiring: it helped them to:	Reinvigorate and renew their interest in educational research Focus on projects previously put ‘on the back burner’ Have time to work on projects
Mentor/mentee relationship		Recognized as an expert in the field	Validation of one’s expertise Informs superiors and peers about their work
Evaluate teaching and education research through a scholarly lens		Increased their knowledge base	Learned new subjects Review of other subjects

The themes shared by both accepted applicant and speaker groups were *community, collaboration, AERI’s organization,* and *motivational/incentivizing*. The theme of *community* appeared frequently in the transcripts. Accepted applicants described their ability to be part of a group that they previously felt they were not qualified to be in. Accepted applicants mentioned they were able to gain self-confidence in their abilities by working with the group, and that they saw that in this diverse group, everyone was focused on the same main goals. Participating in AERI helped these individuals’ perception of the community change as well. As two accepted applicants mentioned:

A: *For me, AERI was life changing…. I used to come to these meetings and I had to sit at the back, I was quiet. I might ask a question or two, but I felt so intimidated. Now, I just feel part of the community. That was one. Just feeling part of the anatomy community, but then also just like, "I can do this," and then having the goals and chunking it out, I made so much progress. My larger project, I can see the finish line. It changed my self-confidence, it changed my perception of the community. It's just been the best thing that I could have done.*

A: *I think one of the greatest things about it was we all walked into a room with basically ... We all wanted the same thing, and we looked around and here was this diverse group of people who all wanted the same thing too.*

The speakers also highlighted the benefit of community, but through a slightly different lens. One individual mentioned how being an educational researcher at their institution was a bit isolating, but AERI allowed this person to get together with other educational researchers and be a part of a bigger group. The speakers described the community as being both the mentors and the accepted applicants, and that all individuals in the community brought something to the group and provided different ways of thinking about the topics. As one speaker noted:

S: *Where I am, it can be kind of isolating if you're an educational researcher, because there aren't any, but it was really nice to be a part of a community, again, I don't get that throughout the year very much, and so, to have a full week to actually interact with other people, like have some other way of thinking, or share ideas and hear what other people are doing is really, really nice.*

Both accepted applicants and speakers appreciated the potential for *collaboration* at AERI. This theme had two shared subthemes: people appreciated the collaboration *a) with other mentees* as well as collaboration *b) with other mentors.* Accepted applicants further noted the collaboration *with other twitter followers,* while the speakers mentioned *multi-institutional collaborations.* The speakers noted that a 6-institution educational research collaboration developed as a result of participating in AERI.

A third theme shared by accepted applicants and speakers was *AERI’s organization*. Specifically, they mentioned that the layout and format of AERI allowed them to focus – it allowed them to goal-set, prioritize steps, and organize their workflow for success. In other words, AERI helped them set up goals that translated to actionable items and a deliverable product. A comparison of accepted applicant and speaker quotes illustrates this commonality:

A: *I'm into medical education and research, but in an unorganized way almost for 10 years, but when I came to AERI, my whole concept, they became consolidated, and I could use them when I reached back home in India, and completed my project also. I did project within those nine months.*

S: *…I set more specific goals and was able to complete some of it, so just taking the time at AERI to devote to setting goals and prioritizing was helpful to build some momentum, even with the lack of time for the rest of the year.*

Both groups of AERI participants found AERI *motivational/inspiring.* For the accepted applicants, AERI encouraged them to expand their knowledge base, and complete their projects. It also inspired them to present and publish their education research projects. For some, participating in AERI inspired them to pursue additional education on their own:

A: *It [AERI] also reminded me that I love statistics and now I'm taking classes.*

A: *If I hadn't gone through the summer, I wouldn't have put in two abstracts that got accepted for here [Experimental Biology].*

The speakers similarly felt AERI was *motivational/inspiring*, but they also focused on the fact that AERI helped reinvigorate and renew their interest in educational research. The institute gave them time to focus on projects they had put ‘on the back burner’ due to lack of time during the rest of the year. As one speaker commented:

S: *Going to AERI made me realize all of these projects I had on the back-burner that I need to get done to help me reinvigorate my interest in kind of the smaller projects I had and realize that I can do a few small-scale things, it doesn't have to be the huge things that I have on the list, right away, but you can chip away at them.*

While the themes of *community, collaboration, AERI’s organization,* and *motivational/incentivizing* were seen in both speakers and accepted applicants, there were a few themes that appeared in one group, but not the other. For example, themes that emerged from discussion with the accepted applicants included the *mentor/mentee relationship* and *evaluating teaching and education research through a scholarly lens.* Accepted applicants consistently spoke positively about their relationship with their mentors. Their mentors helped them think through their project and helped them overcome some roadblocks. Additionally, several noted that now they are evaluating both their teaching as well as research presentations through a scholarly lens. AERI provided them with the tools for that systemic evaluation. One accepted applicant noted this difference in view as they were attending the Experimental Biology 2018 meetings:

A: *This is my first meetings, post AERI, and I'm evaluating a lot of the sessions very differently this year based on some of the stuff I was exposed to, and really seeking rigor. ...being really appreciative when things are done well, and being able to recognize the difference between a well done talk or a well done study in an educational study outside of the more traditional basic sciences, which I'm more comfortable with. That's been a surprise. I didn't even realize it until yesterday's sessions.*

Themes seen only with the AERI speakers included being *recognized as an expert* in the field and *increased their knowledge base.* The speakers found the act of participating in AERI to be validating for their expertise, and it helped inform others (such as superiors and peers) about their work. In addition, many of the speakers mentioned they learned a lot from the sessions and the sessions served as a good review for them.

#### Obstacles to Performing Educational Research

Table 4 lists the themes and codes that emerged from responses to the question “What are your obstacles to performing education research – are they different from what you mentioned on the 6^th^ month survey? Is there anything that AAA or AERI could do to help with those obstacles?” Codes and themes were compared between the accepted applicant group (A) and the invited speaker/mentor group (S). Both accepted applicants and speakers agreed that *time* was one of the biggest obstacles, and thus time was the primary theme. This theme had the following subthemes: *general* time restrictions, *working with collaborators,* and *teaching-education research balance.* Both groups discussed general time constraints, the timing issues when working with collaborators (and getting projects done in a timely fashion). As members from each group noted:

A: *Timing remains an obstacle where, especially again with collaborative stuff, you're all on different schedules and different institutions.*

S: *I think the biggest thing for me is watching certain people be really excited but then not follow through. I think I find that all the time in education research.*

**Table 4:** Thematic Analysis Codebook for “What are your obstacles in performing education research?

Accepted Applicants (A)		Speakers (S)	
**Theme**	**Subtheme**	**Theme**	**Subtheme**
Time	General Working with collaborators Teaching-education ••research balance	Time	General Working with collaborators Teaching-education research balance
IRB	Within institution Across multiple institutions		
Institution	Dean Department IT (instructional technology)		
Culture of devaluing medical education scholarship			

Within this general theme of time, both groups also discussed the conflict between teaching responsibilities and education research responsibilities – that when time is limited, focus goes to one’s teaching first (as that is the more time-sensitive public endeavor). As one accepted applicant noted about education research:

A: *But it's [education research] one of those things that's just very easy to kick the can down the road and not do it today, because you have to prepare for changing an exam or something else. So I think that's probably very common of people who, you know, they're educators first and researchers second or third or fourth.*

One speaker noted that if he does not finish his education research projects and doesn’t get tenure, that ‘failure’ is his alone, and not a failure to his students. Thus, his focus tends to be on teaching:

S: *If you don't prep for your lecture, and if you're not ready for those questions, that is such a public failure, and that, for me, more than anything, is the obstacle to my time, is I would much rather go through my lectures and look into the primary literature and make sure I'm ready to answer questions than do the educational research that is important for promotion and tenure, yes, but if I don't get that, that only affects me. If I'm not a good educator, that affects hundreds of students, (and) looks badly upon my department.*

Interestingly, the speakers primarily focused on time as their main obstacle. In contrast, accepted applicants noted other obstacles (which were represented as themes), such as the *IRB*, *institutional obstacles*, and the *culture that devalues medical education scholarship*.

The *IRB* obstacle theme had the following subthemes of *within* institution and *across multiple institutions.* The accepted applicants mentioned frustration with the IRB bureaucracy at their own institution, and unfamiliarity with the appropriate verbiage to use when submitting the paperwork for an education research study. Another accepted applicant expressed frustration with how to navigate IRB requirements across multiple institutions, for a multi-institutional study:

A: *It's one of those things where that's been an impediment in a couple of projects that I've started right now is just realizing, who's going to be the IRB of record? What does that mean for my institution? It's totally in the weeds, but that's all of the studies that I'm part of that have come out from AERI, are all multi institution. That's what we're running up against.*

Interestingly, while the invited speaker group mentioned *multi-institutional collaborations* as a benefit of AERI, this same group did not mention IRB as an obstacle.

The accepted applicant group also noted *institutional obstacles*, which had the subthemes of *Dean, Department,* and *IT* (*instructional technology).* For some accepted applicants, their enthusiasm about their project was lessened when they did not receive support from the Dean or Department (in the form of protected time, or permission to run a study). Some others had projects that required technological aids and their institution’s IT department was unable or unwilling to assist with the development or administration of such aids. As this accepted applicant noted:

A: *The problem was with the IT…. people in IT said, "Oh, we don't have that type of platform." But the bosses stopped me there. It could not continue. The students, they even wanted to contribute during those evaluations because they said that way you can correct some things that you don't know.*

Finally, the accepted applicants noted the theme/obstacle of *the culture devaluing medical education scholarship.* They felt that education research does not get the same credit or time-protected status as bench research does. And as educators, they are expected to perform their education research on top of all their existing responsibilities. As one accepted applicant noted:

A: *I think really that's part of the culture of medical school that values bench top research and allows you to set aside time very specifically for that, whereas as a basic sciences educator, you're just supposed to figure out when you're going to do all of this stuff and sit down and write it up. That time is not protected in any way, but the expectation is still there.*

As previously mentioned, the invited speakers primarily discussed the obstacle of time, and not the other obstacles that the accepted applicants mentioned. It is unclear why these other topics were not mentioned by the speakers though one possibility is that some of these obstacles (such as navigating the IRB) lessened as they gained experience in the field and became more familiar with study design that could be more easily managed. This will be considered further in the Discussion section below.

#### Potential improvements to the anatomy educational research institute

A third question that received substantial feedback was “What are some things we could do to improve future versions of AERI?”. While this question does not relate as well to the other themes discussed above (and thus will be kept brief), the authors would like to mention the suggestions that were offered from both speaker and accepted applicant focus groups and interviews for those who might choose to develop a similar initiative. Among their suggestions were the following:

- Re-tailor the mentor–mentee pairing. Both speakers and accepted applicants suggested re-tailoring the mentor–mentee pairing. Instead of being paired with a mentor or mentee for the entire institute, both groups suggested switching out mentors and mentees partway through the institute.
- Have built-in writing blocks or independent working time. While both groups wanted time to work on their projects, the mentors said they would have appreciated time for them to work on their own projects and collaborations as well.
- Have accepted applicants and speakers report back on their progress about 6 months out. This reporting would encourage individuals to complete their work.
- Have participants develop three different education research questions, instead of just one. Accepted applicants noted that sometimes their original idea was not feasible, and they only learned that fact upon returning to their institution. By developing several questions, there would be another project as a ‘back up.’
- Make AERI a more frequent event. Accepted applicants and speakers were interested in seeing future AERI meetings and more follow up sessions after an existing AERI. These follow-up sessions could be in the form of online modules for self-understanding and tutoring, or perhaps a workshop at an AAA meeting.

### Thirty-month survey

Data from the thirty-month survey continued many of the themes previously described. Twenty-four AERI 2017 participants (38.7%) completed the thirty-month follow-up survey. Of these twenty-four, fourteen were accepted applicants and ten were invited speakers.

#### Teaching or education research experience

Data on different types of scholarly teaching or education research experiences were compared between the thirty-month follow-up survey and the original pre-AERI survey (Table 5). The data demonstrate that the accepted applicants who responded had indeed become much more active in the anatomy education research community. In particular, participation in publicly available scholarship (e.g., posters, platforms, blogs, other publications) had increased substantially. For example, the percentage of accepted applicants who had published their educational research findings in a journal rose from 19.4% pre-AERI to 57.14% on the thirty-month survey. In addition, accepted applicants were also more active in sharing their new education research knowledge with others (e.g., working with their center for teaching and learning (CTL), participating or leading journal clubs, or mentoring others). For example, the percentage of accepted applicants who had mentored a colleague or student on educational research methods rose from 27.8% to 71.43%.

**Table 5:** Comparing experiences prior to and since the Anatomy Education Research Institute (AERI) 2017

	**Prior to AERI 2017** (data from Husmann et al. [4])		**Since AERI 2017**	
**Teaching or Education research experience**	**Invited Speakers** **(*n* = 20)**	**Accepted Applicants** **(*n* = 36)**	**Invited Speakers** **(*n* = 10)**	**Accepted Applicants** **(*n* = 14)**
Led a teaching and learning workshop at my institution	12 (60%)	14 (38.9%)	8 (80%)	5 (35.71%)
Attended (but did not lead) a teaching and learning workshop at my institution	17 (85)	30 (83.3)	10 (100)	11 (78.57)
Led a journal club about education or a reading group on a teaching or educational research topic	14 (70)	10 (27.8)	6 (60)	6 (42.86)
Participated in (but did not lead) a journal club about education or a reading group on a teaching or educational research topic	18 (90)	17 (47.2)	8 (80)	8 (57.14)
Worked with an instructional consultant at my institution's Center for Teaching and Learning	15 (75)	13 (36.1)	4 (40)	11 (78.57)
Tried a new teaching method	16 (80)	34 (94.4)	8 (80)	13 (92.86)
Developed a substantial curricular change at my institution (e.g., implemented/received approval of a new major, changed a medical curriculum from subject based to organ-systems based approach, etc.)	19 (95)	20 (55.6)	6 (60)	9 (64.28)
Conducted classroom research (e.g., collected and analyzed evidence about a new teaching method)	19 (95)	18 (50)	4 (40)	11 (78.57)
Read an online resource (wiki page, blog, website) about a teaching or educational research topic	18 (90)	33 (91.7)	9 (90)	13 (92.86)
Read a peer-reviewed article about science education or educational research	19 (95)	35 (97.2)	10^a^ (100)	14^a^ (100)
Read a book about educational research or the Scholarship of Teaching and Learning	19 (95)	24 (66.7)	9 (90)	10 (71.43)
Attended an educational research session at a professional meeting (e.g., Experimental Biology)	19 (95)	30 (83.3)	10 (100)	12 (85.71)
Presented a poster on educational research findings at a professional meeting	19 (95)	21 (58.3)	10 (100)	9 (64.28)
Gave a platform presentation or presented a workshop on educational research findings at a professional meeting (e.g., Experimental Biology, American Association of Clinical Anatomists annual meeting)	18 (90)	12 (33.3)	10 (100)	7 (50)
Wrote up my teaching or educational research findings for a blog or website	10 (50)	2 (5.6)	6 (60)	3 (21.43)
Applied for a teaching or educational research grant	15 (75)	10 (27.8)	5 (50)	6 (42.86)
Collaborated with at least 2–3 individuals on an educational research project	19 (95)	20 (55.6)	9 (90)	12 (85.71)
Mentored a colleague or student on educational research methods	18 (90)	10 (27.8)	10 (100)	10 (71.43)
Published my educational research findings in a journal	19 (95)	7 (19.4)	8 (80)	8 (57.14)
Reviewed educational research manuscripts for a journal	19 (95)	13 (36.1)	9 (90)	6 (42.86)
Serve on an editorial board for an educational research journal	5 (25)	2 (5.6)	5 (50)	3 (21.43)

^a^All indicated that they had done this 2 + times

#### Goal follow-up

Figure 2 demonstrates the percentage of speakers, applicants, and total participants that completed goals either six months or thirty months after AERI 2017. While not everyone that responded was able to complete their goals, over 70% of applicants completed at least one goal and nearly 43% completed two! This was even better than the goal completion rates of our speakers (60% and 30%, respectively). A lack of time remains one of the most common reasons for not being able to complete goals while a lack of confidence, lack of follow-through by others, and lack of support were also mentioned in more than one survey. Other responses also mentioned changing projects or interests.

#### Obstacles

Twelve applicants and ten speakers responded to our question on obstacles that they have encountered since attending AERI 2017. Obstacles mentioned in the thirty-month follow-up survey correlate well with those seen in Table 1 from the six-month follow-up survey, though the thirty-month data was not added to the table due to its pre-existing complexity. Time remains the most common obstacle mentioned by either speakers (six mentions) or applicants (seven mentions). Also mentioned by numerous responses were: lack of collaborators or lack of consistent collaborators (two mentions by speakers, four mentions by applicants), lack of funding (four mentions by speakers, one mention by applicants), lack of expertise (mentioned by four applicants), lack of support (four mentions by speakers, two mentions by applicants), and issues with IRB (three mentions by applicants).

## Discussion

The results presented here from both surveys and focus groups/interviews continue to provide data on the effects of AERI 2017. Kirkpatrick level one refers to participants’ reactions to their training [1, 2]. In our previous manuscripts [3, 4], we demonstrated that the initial reactions to AERI 2017 were very positive. The present research provides support for the continuation of this positive reaction in both the surveys and in the focus groups. When asking for ten words that participants associate with education research, participants included words that coded as *important* and *innovative* suggesting that they are still viewing the field in a positive manner. In addition, the theme of *AERI’s organization* under the perceived benefits of AERI mentioned during the focus groups/interviews also demonstrates a continuation of this positive reaction.

### Lasting effects of the Anatomy Education Research Institute (AERI) 2017

These results also provide evidence that AERI 2017 has increased participant knowledge and contributed to longer term behaviors, as desired with Kirkpatrick levels two and three respectively [1, 2]. The data seen here show a sustained or continued increase in knowledge from that previously reported [4]. This was seen for both the accepted applicants and the invited speakers, further confirming previous research by Steinert [8, 13, 14] that indicated a benefit in knowledge for both mentors and mentees. Thus, our qualitative research demonstrates generalizability and transferability to that of Steinert. This trend was also felt by the speakers themselves, as evidenced with the theme of *increase their knowledge base* that was seen in the speaker focus groups and interview as part of the benefits of AERI 2017.

Behavioral changes were also evidenced through both the surveys and the focus groups and interviews. The survey demonstrated substantial progress towards the goals that were identified during AERI, as over ninety percent of respondents indicated that they had started work on these goals. By thirty months after AERI 2017, accepted applicants reported completing more of their goals than even the speakers had with roughly two-thirds of respondents completing at least one goal and over one-third completing at least two goals. Additional follow-up work will be necessary to continue monitoring this progress.

Data presented on teaching and educational research experience also provide evidence of behavioral change. In particular, the accepted applicants demonstrated substantial increases in publicly available scholarship and in sharing their educational research information with others around them (e.g., mentoring). Both outcomes also further broaden the impact of AERI to individuals who are exposed to the scholarship produced by our AERI attendees or who benefit from the personal guidance that AERI attendees provide to their novice colleagues, thus further increasing the impact of AERI 2017 on the larger anatomy and/or medical education communities. Thus, this may also be considered some early data representing Kirkpatrick’s fourth level of evaluation [1, 2] and illustrating an early positive effect on the larger field of anatomy/medical education.

In addition to the survey data, both speakers and accepted applicants commented on one of the benefits of AERI that was coded as *motivating/inspiring*. The example quotes included above demonstrate that one effect of this motivation was to complete their educational research projects. Furthermore, the code of *evaluating teaching and education research through a scholarly lens* that was seen in the focus groups/interviews with the accepted applicants also provides evidence that participants are continuing to apply the skills and knowledge from AERI well beyond the end of the institute. This work further demonstrates the lasting effects that have occurred from AERI 2017.

### Need for sustained community

While the above results demonstrate the positive effects of AERI 2017, there is still much that would be beneficial to sustaining these effects. Both the surveys and the focus groups/interviews demonstrate the need for a sustained community of practice for educational researchers. This need was seen most directly through the survey results. On the survey question regarding obstacles, the need for *collaboration* was mentioned by both speakers and accepted applicants. This code was even more common on the 6-month follow-up survey than it had been on previous surveys (Table 1) and was still being mentioned at thirty months. In addition, the code of *community* saw a substantial decrease on the question that asked participants to provide ten words that they associated with educational research. While this code was still used on the 6-month follow-up surveys, it was not nearly as prevalent as it had been for the immediate post-AERI survey.

While these codes point to the need for a more sustained community directly, the present authors would like to suggest that a sustained community would also have the potential to counteract a number of the other obstacles that were identified here. One example would be that a sustained community could function as a buffer against the devaluing of the field. This devaluing was seen through the surveys as both *non-rigorous perceptions* and *lack of respect* codes saw an increase in the ten words associated with educational research question from the post-AERI surveys. In addition, the theme of *culture devaluing medical education scholarship* was also seen in the accepted applicant focus group/interview and has been noted previously in the literature [17]. Finally, the thirty-month follow-up surveys also reported a lack of support being felt by respondents.

Other noted obstacles that may benefit from a sustained community may be seen through the accepted applicants focus group/interview as well. Both codes for *IRB* (also seen as an obstacle on the 6-month survey) and *institutional* roadblocks may benefit from community. Lack of expertise and issues with IRB were also noted in the thirty-month follow-up surveys. As these roadblocks were not noted by the invited speakers focus group/interview or on the invited speakers’ thirty-month follow-up surveys, novice educational researchers may be better able to navigate these roadblocks if they have better access to more experienced members of the field.

Finally, the greatest obstacle to educational research that was noted by both invited speakers and accepted applicants was *time*. This was by far the most common obstacle listed by both speakers and accepted applicants on the survey, was mentioned by both groups in the focus groups/interviews, and was the only major obstacle discussed by the invited speakers. Though not the first instance in which time was noted as a significant obstacle for medical science educators [17], these results suggest that this is a major limiting factor that does not go away with additional experience in the field. While the presence of a sustained community alone will not produce more time for individuals to work on educational research projects, a community may be able to help provide accountability for progressing on educational research projects. The benefit of this accountability was specifically noted by the focus group/interview participants as one potential improvement for AERI (have participants report back on their progress after six months) and is further supported by previous literature [18, 19].

Previous literature supports the need for a sustained community to support long-term change in higher education behavior. In particular, this has been noted for educational change in science, technology, engineering, and mathematics (STEM) by Henderson and colleagues [20], who completed a meta-analysis of 191 journal articles from 1995 to 2008. They suggest that a one-time experience (e.g., attending AERI) is not enough to produce long-lasting change. Instead, individuals need continued support to maintain these new behaviors. In addition, the need for continued mentoring to sustain faculty development has also been suggested by Irby and O’Sullivan [5], and by Steinert and colleagues [7, 8, 13].

### Options for development of sustained community

So how do we develop this sustained community that our AERI participants need? Multiple options already exist. As previously noted [3, 4], AERI was developed based on an earlier model from the American Physiological Society Institute for Teaching and Learning (APS-ITL). The APS-ITL has developed an online Physiology Educators Community of Practice (PECOP) group to help sustain their community [21–23]. Unfortunately, they acknowledge that the majority of the PECOP members do not actively engage with the community [22]. They are working to engage more PECOP members by including leadership roles (e.g., blog editors or thought leaders) and promoting in-person networking events at annual meetings, though the results of these efforts remain to be seen.

AERI could also develop an online community, particularly through the Anatomy Connected discussion site hosted by the American Association for Anatomy (AAA). Though a number of education-related groups already exist on this medium, at present these groups are less active in educational research than they are with more general education topics. In addition, in-person networking or reunion-type sessions could be held at the annual AAA meeting. At present, this has not been done due to a desire not to exclude others interested in educational research who have not been able to attend AERI, though it is possible that these sessions could also welcome other AAA members who are simply interested in AERI, even if they have not yet been able to attend. Including these individuals could also help to encourage future participants to AERI. In fact, following the second round of AERI in July 2022, movement to create an online community like this has already begun.

Another option, with which many participants have already contributed, is through Twitter. As O’Loughlin et al. [3] previously noted, the Twitter platform was very active throughout AERI 2017. Thus, to continue many of these conversations on Twitter seems only natural. Unfortunately, not all AERI participants are involved on Twitter, so this does leave out some proportion of interested individuals. In addition, like the APS-ITL PECOP, the Twitter platform allows for even those participants who are on Twitter to passively view the interactions without necessarily engaging and thus demonstrating the full benefit of the community.

Finally, one additional opportunity may be to continue some mentor/mentee or other small group relationships via periodic online video meetings. These meetings could serve as check-in sessions to keep participants moving towards their goals. Meeting in these smaller groups via video chats could encourage the active participation of all individuals. These groups could meet at times, frequencies, and for lengths determined by the members of each group and could function similar to “research circles” or “writing groups”, which have previously been shown to help encourage and maintain faculty productivity via goal setting and accountability [18, 19]. An additional benefit to these online video chats would be that the financial and temporal resources necessary for these groups would be minimal but the engaged, individual interactions could still be continued. Following the second round of AERI in July 2022, this proposal was initiated by encouraging each mentoring group to schedule a follow-up online video meeting to occur within the next six months before the participants left the institute.

### Limitations

As with all research, a certain number of limitations to this work must be acknowledged. One of the limitations that was encountered with the follow-up surveys and focus groups/interviews that have been presented here was the limited amount of time that had passed since AERI 2017. With additional time, it is entirely possible that more speakers and applicants may be able to complete the goals that were set at AERI 2017. However, the authors felt that thirty months was a reasonable amount of time for people to show at least some behavioral changes.

Another limitation is our response rate of less than 40% of participants. While this response rate is not ideal and does leave some room for skewed results, as it is likely that those who did participate may have had more positive experiences, the authors nonetheless believe that these data represent a large enough sample to be relevant. It must further be acknowledged that the thirty-month follow-up surveys were distributed as the Covid-19 pandemic was beginning in the United States. These outside issues may have also impacted our response rates for that survey. At the same time, the need for a community of practice among educators and educational researchers was only further demonstrated throughout the ensuing public health crisis.

Finally, as our reflexivity statement addressed, we recognize that having the co-organizers of AERI facilitate the focus groups and interviews likely impacted the feedback given. While all three organizers attempted to convey to participants the need for honest and complete feedback, there is always the possibility that participants felt the need to respond positively to AERI in order to avoid conflict. However, the positive responses also provided in the online surveys (which provide some anonymity) do lend support to the idea that these positive responses were genuine.

## Conclusions

The results of the present study provide continued evidence that the Anatomy Education Research Institute (AERI) 2017 was successful on Kirkpatrick and Kirkpatrick’s [1, 2] Levels of Evaluation 1, 2, and 3. The first level of evaluation was previously demonstrated [3, 4], though continuation of participants’ positive reactions to AERI 2017 were also suggested in both surveys and focus groups/interviews. The second level of evaluation assesses learning from the training. While Husmann et al. [4] demonstrated that learning had occurred, the surveys presented here further demonstrate that this learning was sustained. Finally, the third level of evaluation assesses behavioral changes related to the training, which were demonstrated on the surveys through progress towards completing the educational research goals set during the institute. In addition, the application of the new knowledge and skills formed at AERI were also demonstrated in the focus groups and interviews with the accepted applicants and continuing experiences in teaching and educational research also suggest that participants are sharing their knowledge through scholarly publication and mentorship with those around them.

While the evaluations of AERI 2017 have been largely positive, the need has also been demonstrated for a sustained anatomy education research community to allow for additional collaboration, buffering against devaluing of the field, assistance with IRB and institutional roadblocks, and potential accountability to assist with the most common obstacle: time. Multiple options for this sustained community were provided above, including some that have already been initiated.

## Supplementary Information

**Additional file 1.** AERI 2017 focus group semi-structured protocol.

**Additional file 2.** AERI 2017 30-month follow-up survey.

**Additional file 3.** AERI 2017 6-month follow-up survey.

## Data Availability

The datasets used and/or analyzed during the current study are not publicly available due to the personal nature of the qualitative data but are available from the corresponding author on reasonable request.
