# Supplementary material for: Do the benefits continue? Long term impacts of the Anatomy Education Research Institute (AERI) 2017
Source: BMC Med Educ. 2022 Nov 24;22:810. doi: 10.1186/s12909-022-03883-w (PMC9694568; doi:10.1186/s12909-022-03883-w)
Supplement: Supplementary file 1 — Additional file 1. AERI 2017 focus group semi-structured protocol. [file 12909_2022_3883_MOESM1_ESM.docx]

AERI focus group questions:

1. What have you found most beneficial from what you learned at AERI?
2. What obstacles have you run into while trying to complete your AERI projects?
   1. Lack of institutional/departmental support - how is it perceived/recognized (i.e., how overt is it) and how have people found to work through/around it?  Anything that we or AAA could do to help?
   2. lack of time - how to work through/around?  Anything that we or AAA could do to help?
3. Looking back now, did AERI meet the goals that you had for it when you applied or arrived?
   1. How do people decide which projects to pursue? (A number of people stated that they didn’t meet their goals bc other projects came up)
4. What do you wish you had known prior to attending AERI?
5. How could we improve AERI?
6. if AeRI 2019 happens, would people want to attend again (assuming a fairly similar format) or would that be worthless (<— find a better word)?
7. Additional potential question: who would they think that AERI is best suited for (I.e., who should we target)?

If there’s time: how to pursue collaborations outside of your institution?
