# Supplementary material for: Do the benefits continue? Long term impacts of the Anatomy Education Research Institute (AERI) 2017
Source: BMC Med Educ. 2022 Nov 24;22:810. doi: 10.1186/s12909-022-03883-w (PMC9694568; doi:10.1186/s12909-022-03883-w)
Supplement: Supplementary file 2 — Additional file 2. AERI 2017 30-month follow-up survey. [file 12909_2022_3883_MOESM2_ESM.docx]

AERI 30-mo follow-up

Start of Block: Default Question Block

Q1
You attended the July 2017 Anatomy Education Research Institute (AERI) in Bloomington, IN and responded to the end of conference survey. The purposes of this follow-up survey are to: Gather your retrospective perspectives on AERI 2017

Assess to what extent you were able to implement and complete action items related to the 3 big teaching/educational research goals you listed

Learn about potential collaborations that were developed as a result of attending AERI Gather feedback that may be incorporated for future AERI meetings

Evaluate the effectiveness of AERI as a change agent.
This survey should take no more than 10 minutes of your time, and the data you provide us will allow us to plan for potential future conferences. If you have any questions about the survey, please contact Valerie O’Loughlin at vdean@indiana.edu. (Some survey questions were adapted with permission from the American Physiological Society, 2016.)
  
This educational research survey was reviewed by Indiana University IRB. Our educational research project was reviewed by Indiana University IRB.  Our educational research project was classified as Exempt and is listed under IU IRB Protocol # 1704969308.

Q15
Now that nearly 3 years have passed since you participated in AERI, what aspect of AERI do you feel has been most helpful or useful to you? Please explain.

________________________________________________________________

________________________________________________________________

________________________________________________________________

________________________________________________________________

________________________________________________________________

Q16
What are some of the obstacles, challenges or drawbacks you have encountered in performing educational research?

________________________________________________________________

________________________________________________________________

________________________________________________________________

________________________________________________________________

________________________________________________________________

End of Block: Default Question Block

Start of Block: Block 2

Q24 Since the completion of AERI in July 2017, please state to what extent you participated in the following teaching and educational research activities:

|  | Have not done (1) | Have thought about but not done (2) | Have done once (3) | Have done 2-3 times (5) | Have done more than 3 times (6) | Not sure (7) |
| --- | --- | --- | --- | --- | --- | --- |
| Attended (but did not lead) a teaching and learning workshop at my institution (1) |  |  |  |  |  |  |
| Lead a teaching and learning workshop at my institution (2) |  |  |  |  |  |  |
| Participated in (but did not lead) a journal club about education or a reading group on a teaching or educational research topic (3) |  |  |  |  |  |  |
| Lead a journal club about education or a reading group on a teaching or educational research topic (4) |  |  |  |  |  |  |
| Worked with an instructional consultant at my institution's Center for Teaching and Learning (5) |  |  |  |  |  |  |

Q44 Since the completion of AERI in July 2017, please state to what extent you participated in the following teaching and educational research activities:

|  | Have not done (1) | Have thought about but not done (2) | Have done once (3) | Have done 2-3 times (4) | Have done more than 3 times (5) | Not sure (6) |
| --- | --- | --- | --- | --- | --- | --- |
| Tried a new teaching method (1) |  |  |  |  |  |  |
| Developed substantial curricular change at my institution (e.g., implemented/received approval of a new major, changed a medical curriculum from subject based to organ-systems based approach, etc.) (2) |  |  |  |  |  |  |
| Conducted classroom research (e.g., collected and analyzed evidence about a new teaching method) (3) |  |  |  |  |  |  |
| Read an online resource (wiki page, blog, website) about a teaching or educational research topic (4) |  |  |  |  |  |  |
| Read a peer-reviewed article about science education or educational research (5) |  |  |  |  |  |  |
| Read a book about educational research or the Scholarship of Teaching and Learning (6) |  |  |  |  |  |  |

Q45 Since the completion of AERI in July 2017, please state to what extent you participated in the following teaching and educational research activities:

|  | Have not done (1) | Have thought about but not done (2) | Have done once (3) | Have done 2-3 times (4) | Have done more than 3 times (5) | Not sure (6) |
| --- | --- | --- | --- | --- | --- | --- |
| Attended an educational research session at a professional meeting (e.g., Experimental Biology) (1) |  |  |  |  |  |  |
| Presented a poster on educational research findings at a professional meeting (or have submitted an abstract to do so in the near future) (2) |  |  |  |  |  |  |
| Gave a platform presentation or present a workshop on educational research findings at a professional meeting (or submitted an abstract to do so in the near future) (3) |  |  |  |  |  |  |
| Wrote up my teaching or educational research findings for a blog or website (4) |  |  |  |  |  |  |
| Applied for a teaching or educational research grant (5) |  |  |  |  |  |  |

Q46 Since the completion of AERI in July 2017, please state to what extent you participated in the following teaching and educational research activities:

|  | Have not done (1) | Have thought about but not done (2) | Have done once (3) | Have done 2-3 times (4) | Have done more than 3 times (5) | Not sure (6) |
| --- | --- | --- | --- | --- | --- | --- |
| Collaborated with at least 2-3 individuals on an educational research project (1) |  |  |  |  |  |  |
| Mentored a colleague or student on educational research methods (2) |  |  |  |  |  |  |
| Submitted (but have not yet published) my educational research findings to a journal (3) |  |  |  |  |  |  |
| Published my educational research findings in a journal (4) |  |  |  |  |  |  |
| Reviewed an educational research manuscript for a journal (5) |  |  |  |  |  |  |
| Served on an editorial board for an educational research journal (6) |  |  |  |  |  |  |

End of Block: Block 2

Start of Block: Goal 1 Block

Q46 As a reminder, here are the 3 teaching/educational research goals specific to you or your situation that you planned to follow through on as a result of attending AERI and interacting with the speakers and attendees.  


Goal 1: ${e://Field/Goal%201}


Goal 2: ${e://Field/Goal%202}


Goal 3: ${e://Field/Goal%203}

Q47 What was your planned goal #1?  Please retype here:

________________________________________________________________

Q48 Did you start actions leading to goal #1?

- Yes (1)
- No (2)

Display This Question:

If Did you start actions leading to goal #1? = No

Q70 Please explain why you were unable to start this goal.

________________________________________________________________

Display This Question:

If Did you start actions leading to goal #1? = Yes

Q49 Did you complete goal #1?

- Yes (1)
- No (2)

Display This Question:

If Did you complete goal #1? = Yes

Q71 Please state the results and outcomes from completing this goal.

________________________________________________________________

Skip To: End of Block If Condition: Please state the results an... Is Displayed. Skip To: End of Block.

Display This Question:

If Did you complete goal #1? = No

Q50 Please explain why you were unable to complete this goal.

________________________________________________________________

________________________________________________________________

________________________________________________________________

________________________________________________________________

________________________________________________________________

Q51 Are you still planning to complete this goal in the future?

- Yes (1)
- No (2)

End of Block: Goal 1 Block

Start of Block: Goal 2 Block

Q55 As a reminder, here are the 3 teaching/educational research goals specific to you or your situation that you planned to follow through on as a result of attending AERI and interacting with the speakers and attendees. 


Goal 1: ${e://Field/Goal%201}


Goal 2: ${e://Field/Goal%202}


Goal 3: ${e://Field/Goal%203}

Q56 What was your planned goal #2?  Please retype here:

________________________________________________________________

Q57 Did you start actions leading to goal #2?

- Yes (1)
- No (2)

Display This Question:

If Did you start actions leading to goal #2? = No

Q72 Please explain why you were unable to start this goal.

________________________________________________________________

Display This Question:

If Did you start actions leading to goal #2? = Yes

Q58 Did you complete goal #2?

- Yes (1)
- No (2)

Display This Question:

If Did you complete goal #2? = Yes

Q73 Please state the results and outcomes from completing this goal.

________________________________________________________________

Skip To: End of Block If Condition: Please state the results an... Is Displayed. Skip To: End of Block.

Display This Question:

If Did you complete goal #2? = No

Q59 Please explain why you were unable to complete this goal.

________________________________________________________________

________________________________________________________________

________________________________________________________________

________________________________________________________________

________________________________________________________________

Q60 Are you planning to complete this goal in the future?

- Yes (1)
- No (2)

End of Block: Goal 2 Block

Start of Block: Goal 3 Block

Q61 As a reminder, here are the 3 teaching/educational research goals specific to you or your situation that you planned to follow through on as a result of attending AERI and interacting with the speakers and attendees.  


Goal 1: ${e://Field/Goal%201}


Goal 2: ${e://Field/Goal%202}


Goal 3: ${e://Field/Goal%203}

Q62 What was your planned goal #3?  Please retype here:

________________________________________________________________

Q63 Did you start actions leading to goal #3?

- Yes (1)
- No (2)

Display This Question:

If Did you start actions leading to goal #3? = No

Q76 Please explain why you were unable to start this goal.

________________________________________________________________

Display This Question:

If Did you start actions leading to goal #3? = Yes

Q64 Did you complete goal #3?

- Yes (1)
- No (2)

Display This Question:

If Did you complete goal #3? = Yes

Q77 Please state the results and outcomes from completing this goal.

________________________________________________________________

Skip To: End of Block If Condition: Please state the results an... Is Displayed. Skip To: End of Block.

Display This Question:

If Did you complete goal #3? = No

Q65 Please explain why you were unable to complete this goal.

________________________________________________________________

________________________________________________________________

________________________________________________________________

________________________________________________________________

________________________________________________________________

Q66 Are you still planning to complete this goal in the future?

- Yes (1)
- No (2)

End of Block: Goal 3 Block

Start of Block: Block 5

Q79 Please upload a current copy of your CV.

| 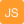 |
| --- |

Q67 If there are any other comments you have about AERI, please state them below.

________________________________________________________________

End of Block: Block 5
