# Supplementary material for: Do the benefits continue? Long term impacts of the Anatomy Education Research Institute (AERI) 2017
Source: BMC Med Educ. 2022 Nov 24;22:810. doi: 10.1186/s12909-022-03883-w (PMC9694568; doi:10.1186/s12909-022-03883-w)
Supplement: Supplementary file 3 — Additional file 3. AERI 2017 6-month follow-up survey. [file 12909_2022_3883_MOESM3_ESM.docx]

You attended the July 2017 Anatomy Education Research Institute (AERI) in Bloomington, IN and responded to the end-of-conference survey. The purposes of this 6-month follow-up survey are to:

- Gather your retrospective perspectives on AERI 2017
- Assess to what extent you were able to implement and complete action items related to the 3 big teaching/educational research goals you listed
- Learn about potential collaborations that were developed as a result of attending AERI
- Gather feedback that may be incorporated for future AERI meetings
- Evaluate the effectiveness of AERI as a change agent.

This survey should take no more than 15 minutes of your time, and the data you provide us will allow us to plan for potential future conferences and apply for grants to fund future AERI conferences. If you have any questions about the survey, please contact Valerie O’Loughlin at [vdean@indiana.edu](mailto:vdean@indiana.edu) . (Some survey questions were adapted with permission from the American Physiological Society, 2016.)

This educational research survey was reviewed by Indiana University IRB. Our educational research project was classified as Exempt and is listed under IU IRB Protocol # ________________

**As a reminder, here is your 3 digit identification # that you listed on your end-of-conference survey:**

**_______**

**Educational research knowledge and beliefs**

1. In the textbox below, please write up to 10 words or phrases you think of when you hear the phrase ‘educational research’. These words MAY or MAY NOT align with the 10 words you wrote on the pre and end of conference AERI surveys. These words may refer to how easy or difficult you think it is to perform educational research, how this research is regarded compared to bench research, your level of knowledge about educational research, etc. Be creative!
2. Now that 6 months has passed since you participated in AERI, what aspect of AERI do you feel has been most helpful or useful to you? Please explain.
3. Now that 6 months has passed since you participated in AERI, was there an event or topic that was **not** discussed at AERI, but you feel should have been a part of the program? (If there was none, please state ‘none’).
4. Now that 6 months has passed since you participated in AERI, what are some of the obstacles, challenges or drawbacks you have encountered in performing educational research?

a.

b.

c.

**Educational activities participation follow-up**

1. Since the completion of AERI 2017, please state to what extent you participated in the following teaching and educational research activities:
   1. Have not done
   2. Have thought about but not done
   3. Have done once
   4. Have done 2-3 times
   5. Have done more than 3 times
   6. Not sure

Attended (but did not lead) a teaching and learning workshop at my institution: ___________________

Lead a teaching and learning workshop at my institution: ________________________

Participated in (but did not lead) a journal club about education or a reading group on a teaching or educational research topic: ______________

Led a journal club about education or a reading group on a teaching or educational research topic: ______________

Worked with an instructional consultant at my Institution’s Center for Teaching and Learning: _______

Tried a new teaching method: __________________

Developed substantial curricular change at my institution (e.g., implemented/received approval of a new major, changed a medical curriculum from subject based to organ-systems based approach, etc.): ____________________________

Conducted classroom research (e.g., collected and analyzed evidence about a new teaching method): __________

Read an online resource (wiki page, blog, website) about a teaching or educational research topic: ______

Read a peer-reviewed article about science education or educational research: _________________

Read a book about educational research or the Scholarship of Teaching and Learning: ___________

Attended an education research session at a professional meeting (e.g., Experimental Biology) ______

Presented a poster on educational research findings at a professional meeting: _______________

Gave a platform presentation or present a workshop on educational research findings at a professional meeting (e.g., Experimental Biology, AACA): __________

Wrote up my teaching or educational research findings for a blog or website: _________________

Applied for a teaching or educational research grant: _________________________________

Collaborated with at least 2-3 individuals on an educational research project: ___________________________

Mentored a colleague or student on educational research methods: _________________________

Submitted (but have not yet published) my educational research findings in a journal: _________

Published my educational research findings in a journal: ___________________________

Reviewed an educational research manuscript for a journal: _____________________

Served on an editorial board for an educational research journal: ______________________

1. As a reminder, here are the 3 teaching/educational research goals specific to you or your situation that you planned to follow through on as a result of attending AERI and interacting with the speakers and attendees. Do NOT change these answers – they are here to remind you of your goals and help you answer the questions below:

7. Goal #1

a. What was your planned goal #1? Retype here: _________________

b. Did you start actions leading to goal #1? (yes or no)

c. Did you complete goal #1 (yes or no)

d. If you were able to complete your goal, please state the results and outcomes. If you were unable to start or complete this goal, please explain why.

e. If you did not complete your goal, are you planning on completing it in the future? If so, please state approximately when you plan to complete the goal.

8. Goal #2

a. What was your planned goal #2? Retype here: _________________

b. Did you start actions leading to goal #2? (yes or no)

c. Did you complete goal #2? (yes or no)

d. If you were able to complete your goal, please state the results and outcomes. If you were unable to start or complete this goal, please explain why.

e. If you did not complete your goal, are you planning on completing it in the future? If so, please state approximately when you plan to complete the goal.

9. Goal #3

a. What was your planned goal #3? Retype here: _________________

b. Did you start actions leading to goal #3? (yes or no)

c. Did you complete goal #3? (yes or no)

d. If you were able to complete your goal, please state the results and outcomes. If you were unable to start or complete this goal, please explain why.

e. If you did not complete your goal, are you planning on completing it in the future? If so, please state approximately when you plan to complete the goal.

10. Were there any additional goals or action items related to educational research that you took, that were NOT listed on your end-of-conference survey?

a. Yes b. No

If yes, please explain in the text box below. If no, say ‘none’

11. If there are any other comments you have about AERI, please state them below.

12. Would you be willing to participate in a follow-up interview? Yes No

If yes, please indicate the best way to reach you to set up an appointment: _________________

Thank you for completing this survey! If you have any questions, please contact Valerie O’Loughlin at [vdean@indiana.edu](mailto:vdean@indiana.edu)
